# Black Crust Complex: Influence of Temperature and Period of Wetness on the Development of Fungi in *Hevea brasiliensis*

**DOI:** 10.3390/plants13131821

**Published:** 2024-07-02

**Authors:** Louyne Varini Santos Dos Anjos, Gabriel Leonardi Antonio, Ivan Herman Fischer, Elaine Cristine Piffer Goncalves, Erivaldo José Scaloppi Junior, Edson Luiz Furtado, Thaís Lopes de Oliveira, Heloísa Noemi Bello, Ana Carolina Firmino

**Affiliations:** 1 Agricultural Engineering and Soil, Faculty of Engineering, São Paulo State University (UNESP), Ilha Solteira 15385-000, SP, Brazil; 2 School of Agriculture, São Paulo State University (Unesp), Botucatu 18610-034, SP, Braziledson.furtado@unesp.br (E.L.F.); 3 São Paulo’s Agency for Agribusiness Technology (APTA), Bauru Reginal, Bauru 17030-000, SP, Brazil; 4 São Paulo’s Agency for Agribusiness Technology (APTA), Colina Center, Colina 14770-000, SP, Brazil; elaine.piffer@sp.gov.br; 5 Center of Rubber Tree and Agroforestry Systems, Agronomic Institute of Campinas (IAC), Votuporanga 15505-970, SP, Brazil; scaloppijr@yahoo.com.br; 6 College of Agricultural and Technological Sciences, São Paulo State University (Unesp), Dracena 17900-000, SP, Brazil; thais.lopes@unesp.br (T.L.d.O.);

**Keywords:** rubber tree, environment, fungus, temperature, humidity

## Abstract

The objective of this work was to evaluate the development of *Davidiella* sp. and its asexual form, *Cladosporium* sp., under different environmental conditions in the rubber tree (*Hevea brasiliensis*). Rubber tree leaves were inoculated with a spore suspension and kept in a humid chamber under different temperatures and wetness periods. The behavior of the fungi was evaluated using a scanning electron microscope (SEM) and an ultraviolet light microscope (UV). In the images obtained in SEM, four hours after inoculation of the fungus, it was possible to verify the germination and penetration of conidia at temperatures of 10 to 20 °C. The formation of conidiophores was verified from six hours after inoculation, indicating that it is in the reproductive period. In the sexual phase, in SEM, from four hours after inoculation, it was possible to verify the formation of small protuberances at temperatures between 10 and 20 °C. These black dots evolve into circular, protruding black spots, like the symptoms of black crust, with apparent spore formation on them. The data obtained from the UV analyses corroborate those from SEM, showing that the fungus has good development in its two phases between temperatures of 20 and 25 °C and that the period of wetness on the leaf can contribute to the initial development of the pathogen.

## 1. Introduction

The rubber tree (*Hevea brasiliensis*) is part of the *Euphorbiaceae* family, with the Amazon region as its center of origin. Its economic importance is linked to the production of latex, from which natural rubber is obtained. Although the rubber tree originates from the Amazon and Brazil has cultivation areas distributed across different states, there is still a need for imports, which lead to negative impacts on the country’s trade balance [1]. Brazil currently accounts for just 1% of world production, which only supplies 40% of its demand [2].

Rubber tree cultivation, in addition to playing a fundamental role in obtaining natural rubber, has great social importance, as it is responsible for generating around several jobs, and is also important from an environmental point of view, as it is a culture that sequesters carbon from the atmosphere, in amounts equivalent to those of a natural forest [3,4,5].

Recently, a disease known as black crust has been causing serious problems for this crop in Brazil. This disease has always been treated as secondary, as it only occurs in a few parts of the Amazon region [6,7]. However, over the last few years, the occurrence has become increasingly frequent, causing damage due to the premature fall of leaves and apical death of branches. In the State of Bahia, studies on this disease indicate a 38% reduction in rubber production between 2017 and 2020 [8].

Until the mid-1990s, black crust was mainly associated with two fungi, *Phyllachora huberi* and *Rosenscheldiella heveae*, which can occur alone or together [6]. Between the years 2022 and 2023, Gomes et al. [9] and Antonio [10], after analyzing this disease in rubber tree samples collected in the states of Minas Gerais and São Paulo, reported that they did not observe the fungi *P. huberi* and *R. heveae* in the stroma of the disease, but rather the presence of the fungi *Davidiella* sp. and its asexual form, *Cladosporium* spp. [9,10]. These authors were able to associate the occurrences of black crust with this genus of fungus through pathogenicity criteria. Therefore, the view on this disease has been reevaluated, and it is more prudent to call it black crust complex, as we know that the disease cycle in the field can involve different species of fungi and their phases.

When addressing the rubber tree/*Cladosporium*–*Davidiella* pathosystem, the disease cycle begins with the release of ascospores from fallen leaves. These ascospores infect newly released leaflets, transitioning into an asexual form and carrying out the secondary cycle of the disease. As the leaflets age and stroma emerge, sexual structures appear. Eventually, the leaflets fall due to disease or natural senescence, serving as the primary inoculum source for the cycle to restart [6,10]. The epidemiology of the disease resembles that of South American leaf blight [11].

The greatest severity of black crust occurs during periods of rainfall followed by successive days of mild temperature [10]. There are still no reports in the literature about which environmental conditions or genetic materials encourage the occurrence of the sexual or asexual forms of the fungus, nor which form is more aggressive.

Work related to the control of black crust in rubber trees is scarce and there are no chemical products registered for its control in this crop in Brazil [12]. Therefore, the use of resistant materials would be the best alternative for managing black crust. Guen, Seguin and Mattos [13] evaluated 200 clones for resistance to black crust in the field. According to these authors, 42 clones proved resistant, being derived from *H. brasiliensis*, *H. benthamiana*. However, the authors report that some resistant materials became susceptible during the evaluation period. This variation in reaction to the disease, according to the authors, is due to the interaction of the genotype with the environment.

The environment can have a strong influence on the expression of resistance in rubber tree clones, making the plant more susceptible or not to attack. Therefore, knowledge of the ideal environmental characteristics for the development of the fungus on different plant varieties, clones or cultivars is of great importance in managing the disease in the field [14,15].

For example, in the case of anthracnose on rubber trees, there are reports that *Colletotrichum gloeosporioides* and *C. acutatum* need relative humidity above 90% for 13 h a day and average temperatures between 26 and 32 °C for the disease to occur severely [6,16]. According to Magalhães and collaborators [17], temperatures between 25 and 35 °C and wet periods of 24 h were favorable for the germination and formation of appressoria of *C. tamarilloi* in resistant and susceptible clones, however, with different degrees of fungal development.

Knowing that the environment has an influence on the development of the disease and due to the scarcity of studies in the literature on the environmental conditions that favor the development of black crust on rubber trees, the present study aimed to evaluate the influence of the temperature and period of wetness on the development of *R. heveae*, in its sexual and asexual form, in rubber tree leaflets, and thus better understand the behavior of this disease in this crop, being able to collaborate in the development of control techniques.

## 2. Materials and Methods

### 2.1. Obtaining Sexual and Asexual Spores and Inoculating Rubber Tree Leaflets under Different Environmental Conditions

This work was developed at the end of 2022 and beginning of 2023. To carry out this experiment, spores of *Davideiella* sp. were used, in their sexual and asexual form, which corresponds to the fungus *Cladosporium* sp. [18,19].

The leaf materials with black crust used to obtain these fungi came from the RRIM 600 rubber tree clone, collected in a commercial plantation, located in the city of Barretos-SP, with symptoms and stroma typical of the fungus.

To obtain the sexual spores (ascospores) and isolate the asexual phase (*Cladosporium* sp.), the methodology described by Gasparotto et al. [6] was adopted. The suspension of ascospores obtained from the stroma on the black crust was used to inoculate rubber tree leaves and also to obtain colonies of *Cladosporium* sp. in petri dishes containing PDA medium [6]. The experiments carried out with sexual and asexual spores were set up separately.

The leaflets of rubber tree seedlings in the intermediate phase of the RIMM 600 clones were detached and disinfested. These were later inoculated with aliquots containing 30 μL of suspension of sexual and asexual spores (10^5^ spores/mL). The inoculated area was demarcated with the help of plastic stickers to delimit the inoculation site on the leaf. As a control, leaflets inoculated only with autoclaved distilled water were used. After spore inoculation, the leaflets were kept in a humid chamber at BOD (Biological Oxygen Demand) at different temperatures (10, 15, 20, 25, 30, 35 and 40 ± 1 °C) in the dark. Circular samples, 5 mm in diameter, were obtained from the inoculated areas at pre-determined time intervals (4, 6, 12, 24, 48 and 72 h) and fixed in “Karnovsky” solution. The samples were then processed for evaluation using a scanning electron microscope (SEM) and ultraviolet light microscope (UV). The experiment was conducted in a completely randomized design with eight replications per treatment.

### 2.2. Evaluation of the Development of Davidiella and Cladosporium in Rubber Leaflets Using SEM and Ultraviolet Light Microscopy (UV)

After removing the samples from the “Karnovsky” fixative, the samples were processed according to the methodology described by Firmino et al. [20] to observe the development of the fungus on the surface of the inoculated rubber tree leaves. These samples were mounted on stubs with double-sided carbon tape and coated with 20 nm gold in a Bal-Tec SCD 050 sputter coater (The equipment was sourced by BAL-TEC Inc, Liechtenstein, Balzers), for analysis in SEM LEO435-VP, located at the Electronic Microscopy Center of the “Luiz de Queiroz” School of Agriculture. (ESALQ), University of São Paulo (USP), Piracicaba, State of São Paulo, Brazil.

Observation of the development of the fungus on the inner part of the leaf was carried out using the technique developed by Celio and Hausbeck [21], with modifications. The samples were removed from the “Karnovsky” solution and after processing, they were treated with Calcofluor White (Sigma^®^, St. Louis, MO, USA). This reagent (approximately 50 µL) was placed on the samples, which, after 1 minute, were washed in distilled water (twice for 30 s) and mounted in distilled water for observation under a UV microscope.

### 2.3. Data Analysis

For the images captured in SEM, a descriptive methodology was adopted, based on the observation of the development of the fungus at different temperatures and periods of wetness, like Magalhães et al. [16].

In the case of the UV microscope analyses, the assessment was of the quantitative type of fungal development, based on the structures produced by the fungus (mycelium and spores) in the inner part of the leaf. These analyses were carried out with the Basic Intensity Quantification with ImageJ software [22]. The average percentage of leaf area affected by the fungus in each environmental condition tested was analyzed using the statistical software Sisvar [23]. Based on these analyses, the best period in which the fungus developed was found and then a regression analysis was carried out to find the temperature range in which the fungus had its greatest development, using the statistical software Sisvar 5.6 version [23].

## 3. Results and Discussion

**Figure 1:**
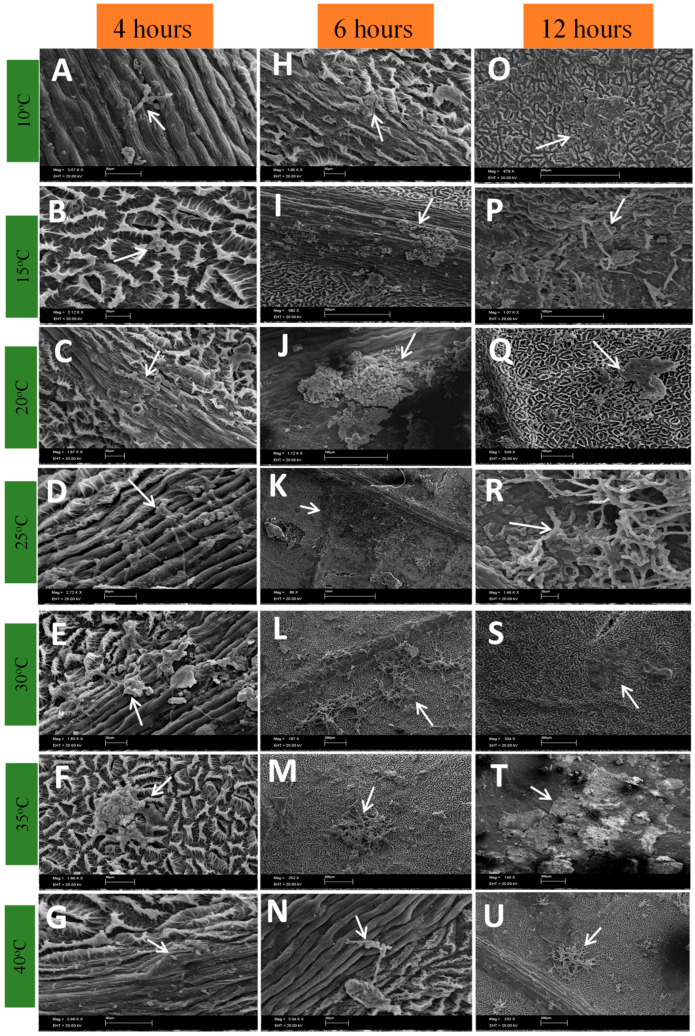
Development of *Cladosporium* sp. on the surface of rubber tree leaves with 4, 6 and 12 h of wetness subjected to different temperatures.

**Figure 2:**
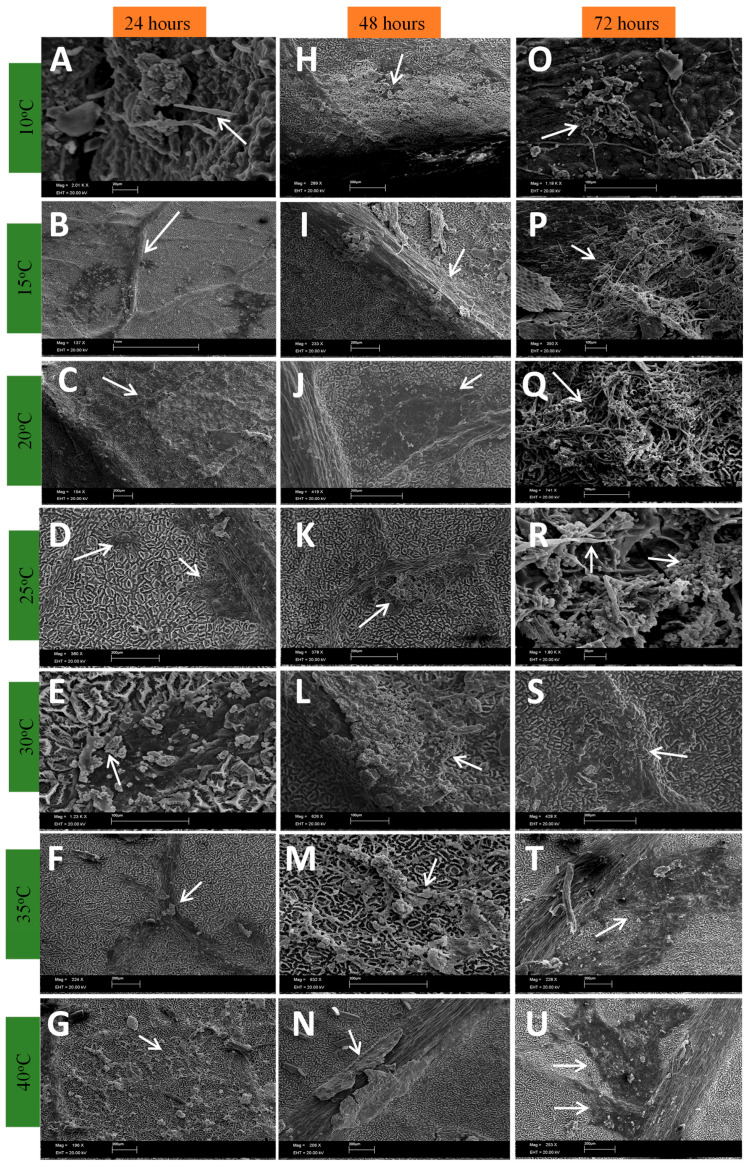
Development of *Cladosporium* on the surface of rubber tree leaves with 24, 48 and 72 h of wetness subjected to different temperatures.

**Figure 3:**
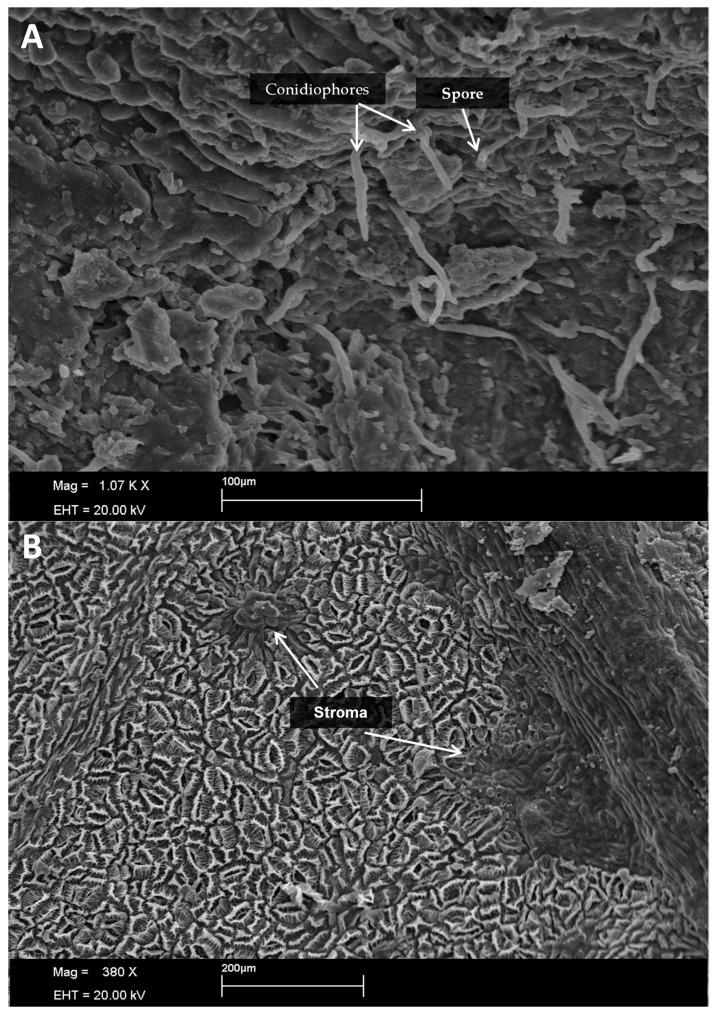
Formation of conidiophores with 12 h of wetness at 15 °C from the inoculation of *Cladosporium* spores and formation of new spores (**A**) and elevation of the surface of infected leaves with possible beginning of stroma formation (**B**).

**Figure 4:**
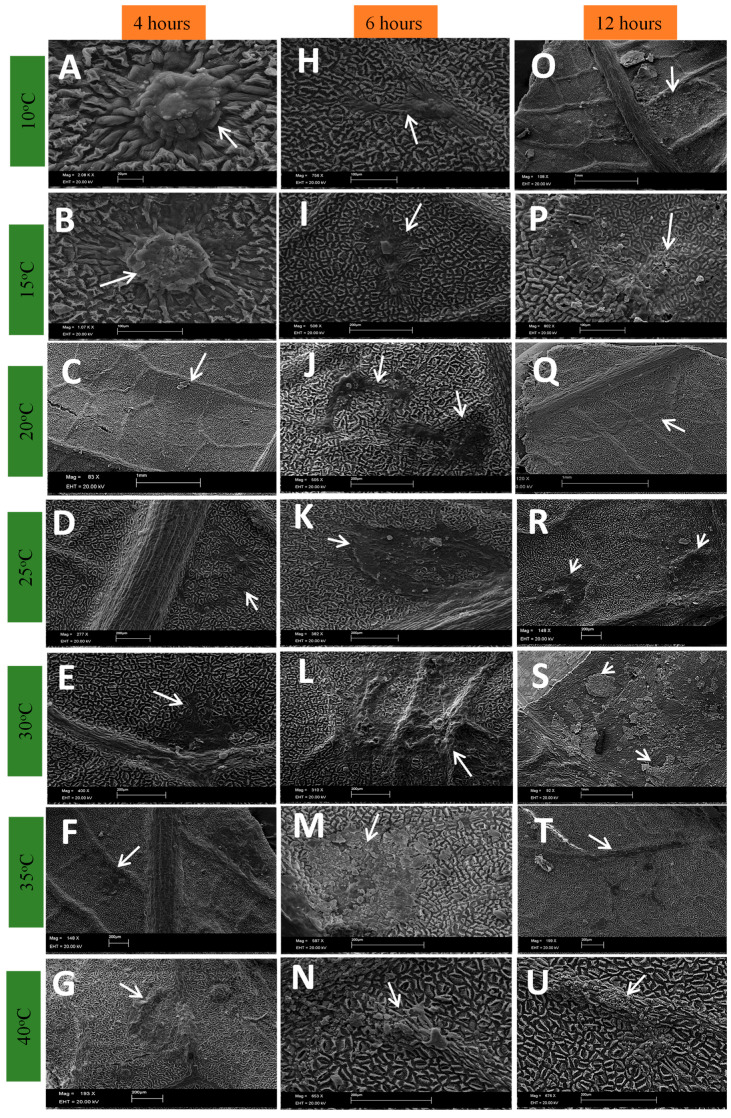
Development of ascospores on the surface of rubber tree leaves in SEM with 4, 6 and 12 h of wetness subjected to different temperatures.

Figure 1, Figure 2, Figure 3 and Figure 4 show the progress of the disease initiated by *Cladosporium* at different temperatures up to 72 h after inoculation. It was observed that *Cladosporium* had a rapid development, as four hours after inoculation of the fungus, it is possible to verify the good development of the fungus at temperatures of 10 to 20 °C. At temperatures of 25 and 30 °C, the development of the fungal mycelium and the beginning of the formation of a crust under the inoculation point are already seen. This onset of crust is also noticeable at 35 °C. At 40 °C, a delay in the development of the fungus was observed in relation to the other temperatures.

During the development of the fungus during the periods analyzed, it was found that in the asexual phase, at temperatures of 10 to 25 °C, from six o’clock onwards, a crust forms that becomes darker over time (Figure 1H–K and Figure 2B,D–J,N,U).

The formation of conidiophores was seen from six hours after inoculation (Figure 1P,R, Figure 2A and Figure 3A), indicating that the fungus is already in the reproductive period as this structure is a conidiogenous cell that has the function of forming new conidia [24]. The formation of new structures such as phialides and spores becomes clearer 72 h after inoculation (Figure 2 and Figure 4), which may indicate that the period of wetness also influences the development of *Cladosporium*.

In Figure 2 and Figure 3B, there is the presence of well-defined protuberances with a rounded shape that may be the beginning of the stroma on the tissue. At temperatures between 30 and 40 °C, the fungus slows down its development, despite the formation of dark plaques at the inoculation points (Figure 2S–U).

In leaflets inoculated with ascospores, specifically *Davidiella*, small protuberances indicative of early stroma formation was observed as early as four hours post-inoculation, at temperatures ranging between 10 and 20 °C (Figure 4A–C). These stromal cells exhibited well-defined, rounded shapes resembling those observed during *Pseudocercospora ulei* development in rubber tree leaves [25].

**Figure 5:**
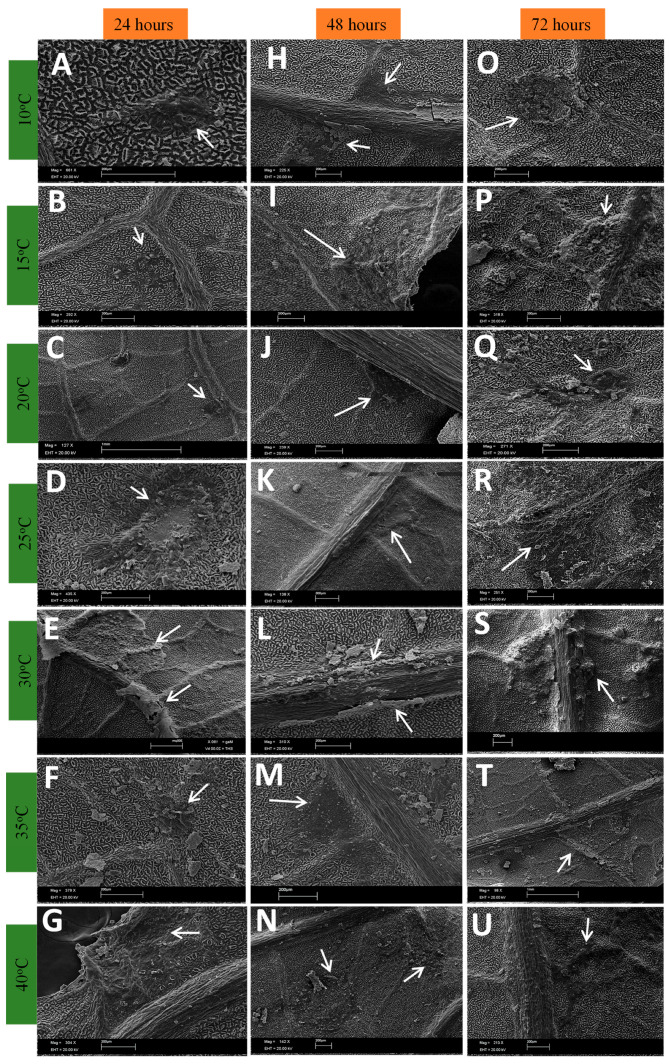
Development of ascospores on the surface of rubber tree leaves in SEM with 24, 48 and 72 h of wetness subjected to different.

**Figure 6:**
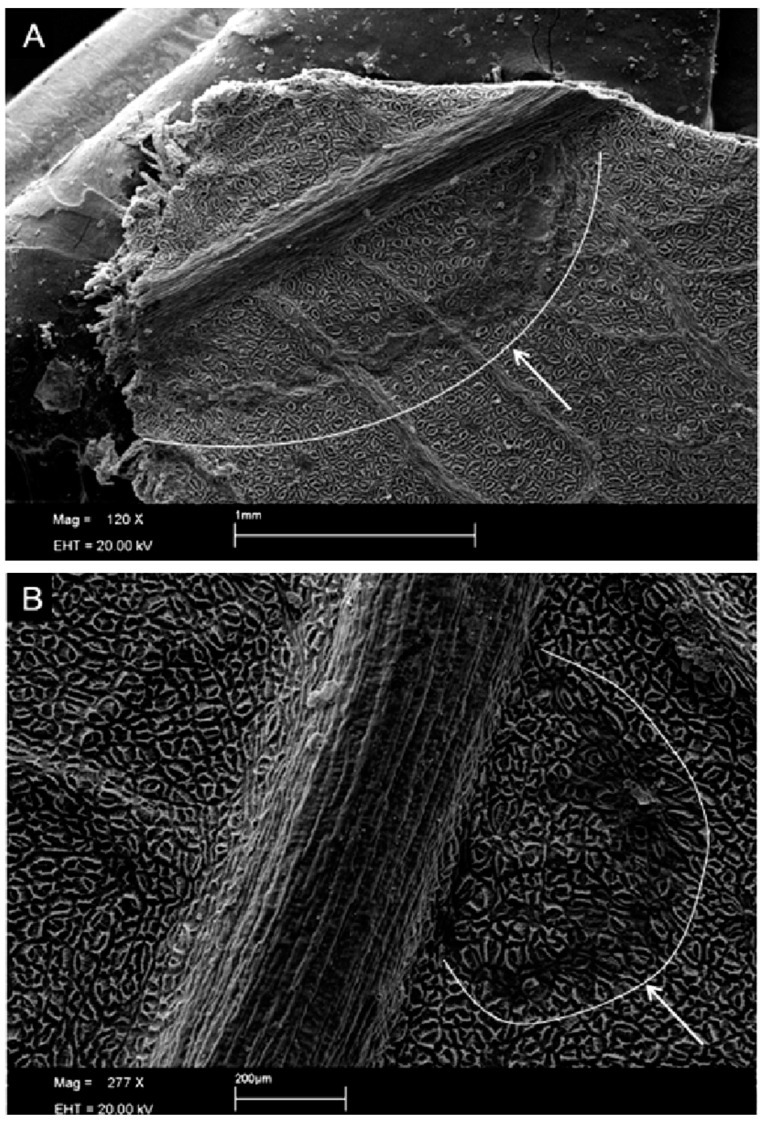
Spots similar to black crust, with circular characteristics, after a 4 h wet period at temperatures of 25 °C (**A**,**B**).

Notably, within the same four-hour post-inoculation timeframe, temperatures between 25 and 30 °C prompted the emergence of black crust-like spots (Figure 4D), along with circular characteristics and the incipient formation of stroma, mirroring field observations. These circular crust formations increased in frequency and area over time (Figure 5 and Figure 6).

At 40 °C, fungal growth slowed, irrespective of the analyzed timeframe (Figure 5N), aligning with data from the analyses of the fungus’s asexual phase. These microscopic observations support findings by Gomes et al. [9], who conducted pathogenicity tests with various *Cladosporium* isolates obtained from black crust stroma. The authors noted challenges in reproducing black crust symptoms on greenhouse-inoculated seedlings due to higher experimental site temperatures. They observed black crust symptoms and early stroma formation approximately 120 days post-inoculation, coinciding with milder regional temperatures, particularly at night. This underscores temperature’s pivotal role in fungal development and symptom expression in plants, potentially affecting the pathogen’s latency period. Higher temperatures seem to retard its development, as also noted by Gasparoto et al. [6].

**Figure 7:**
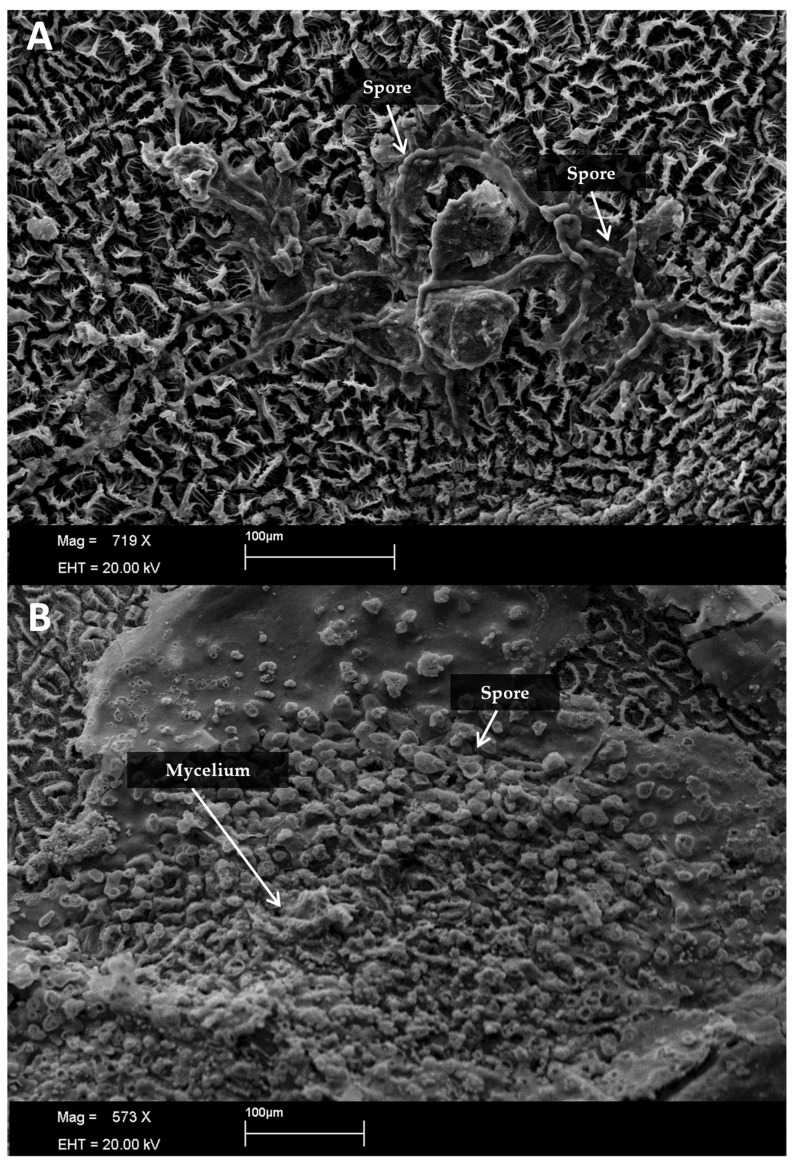
Observation of the formation of *Cladosporium* spores 24 h after inoculation at 20 °C on the surface of rubber tree leaves inoculated with *Davidiella* sp. (**A**,**B**).

Six hours post-inoculation, initial spore and mycelia formation on nascent stroma were observed, persisting across evaluated periods, particularly at temperatures ranging from 20 to 30 °C (Figure 4, Figure 5, Figure 6 and Figure 7). Sporulation following *Davidiella* inoculation revealed conidia akin to those of *Cladosporium* (Figure 7).

**Figure 8:**
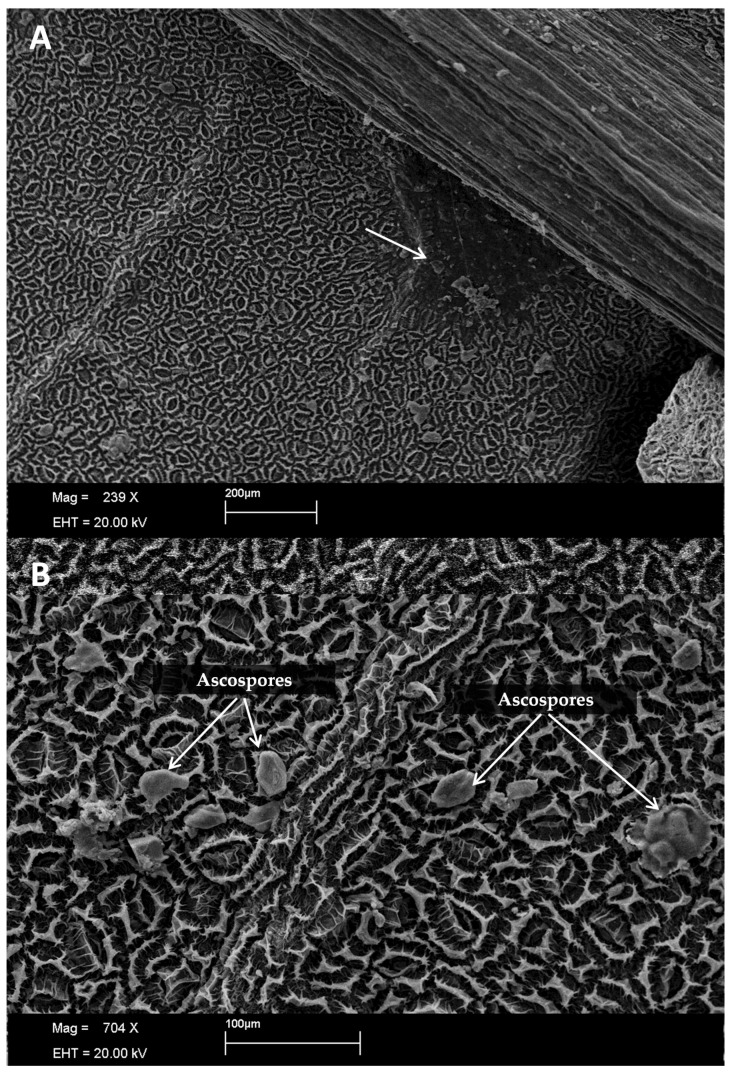
Observation of ascospores 48 h after inoculation at 20 °C on the surface of inoculated rubber tree leaves (**A**,**B**).

Ascospores became more prevalent from 48 h post-inoculation (Figure 8), akin to observations by Gasparotto et al. [6] regarding *P. ulei*, where initial stroma accommodates spermatic depressions resembling pycnidia, progressing to ascogenesis and ascospore maturation. This behavioral resemblance between the two fungi likely stems from their shared family, *Mycosphaerellaceae*.

The results obtained from the inoculation of ascospores indicate that the different symptoms observed in the field may be due to the phase this fungus is in, where the formation of more rounded plaques is seen, like dark spots with no or little elevation with the development of the fungus in the case of asexual spores (*Cladosporium*) and symptoms of crusting in circles with stroma, which appears to be developed by infections initiated by ascospores (*Davidiella*). This behavior has already been reported for leaf blight, caused by *P. ulei* on rubber trees [6]. It has even been shown that in the case of leaf blight, the ascospores of the sexual phase (*P. ulei*) and the conidia of the asexual phase of this fungus (*Fusicladium*) are part of a single cycle, forming a fungal complex, where the ascospores demonstrated play an essential role in the perpetuation of the disease outside the growth of the host or in times unfavorable to the development of the pathogen, and conidia contribute to the resumption of epidemics and the spread of the disease over long and short distances [11,26]. 

Following this line of reasoning and based on the results obtained from the inoculation of *Davidiella* and *Cladosporium*, it is possible that black crust has the same behavior as leaf blight, involving a complex of fungi, with the production of sexual spores and asexual spores that play a determining role in the survival and spread of the disease. This may explain the fact that it was found that samples treated with *Cladosporium* spores present larger leaf areas attacked (Table 1 and Table 2), as this phase would be linked to the spread of black crust.

**Figure 9:**
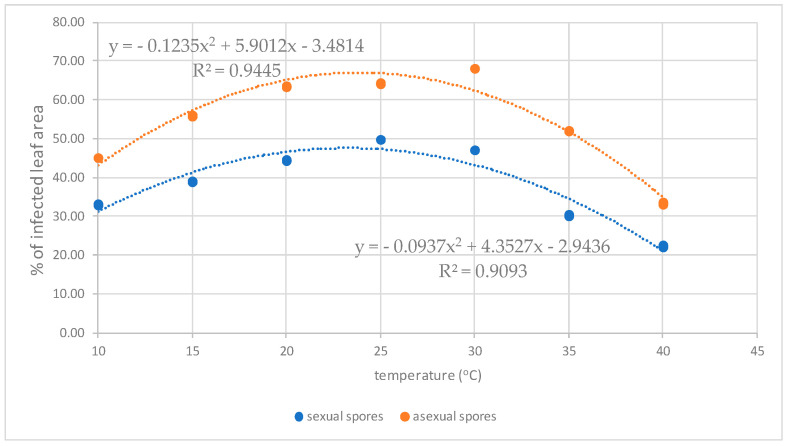
Average percentage of infected leaf area of inoculated samples subjected to different temperatures under a wet period of 72 hours after inoculation.

**Figure 10:**
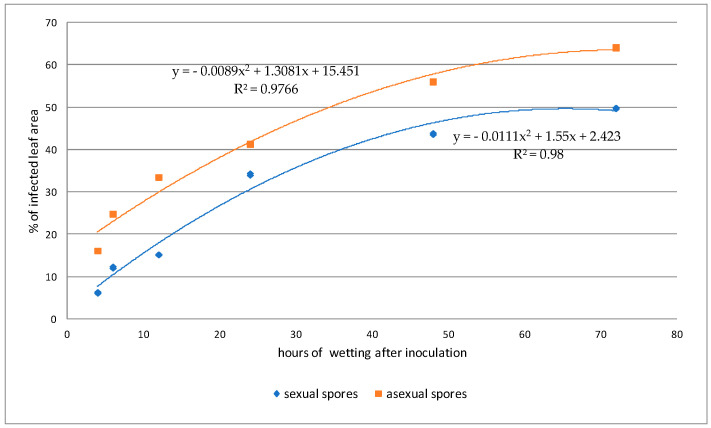
Average percentage of infected leaf area of samples inoculated at different periods of wetness at a temperature of 25 °C.

According to the analyses carried out using UV light microscopy, the fungus had the same behavior reported in the SEM analyses, and it was verified that the best temperatures for the development of both *Cladosporium* and *Davidiella* are close to 20 to 25 °C and that the increase in fungal structures was proportional to the period of wetness (Table 1 and Table 2 and Figure 9 and Figure 10). This information may cause some concern, as two other pathogens of great importance in the rubber tree, *P. ulei* and *Colletotrichum*, also have good development in these environmental conditions, which may worsen the situation of this crop in different Brazilian regions, including the escape zones of rubber of the *P. ulei*.

For example, the southern region of the State of Bahia is part of the “marginal range” of occurrence of *P. ulei*, that is, it is an area where clones resistant to this pathogen are used, since the climate, high atmospheric humidity with occurrence at least 13 h a day with an RH greater than 95% and an average monthly temperature of between 20 and 26 °C, is favorable for the disease to attack. In this region, we have records of a 38% reduction in rubber production between 2017 and 2020 in these clones, due to the attack of the black crust, as it found a favorable environment and a susceptible host [8].

Another example is what happens in the State of São Paulo. In this case, black crust frequently occurs in conjunction with anthracnose, caused by *Colletotrichum*. This may be due to several factors, which we can highlight (i) the predominance of the planting of clone RRIM600, which is susceptible to both diseases [16]; (ii) favorable environment for both pathogens, as, like black crust, anthracnose-causing pathogens develop well in temperature ranges between 20 and 30 °C and their development is directly proportional to the period of leaf wetness [16] and (iii) the lesions caused by the black crust end up serving as an opening for the entry of *Colletotrichum* [6]. This makes it clear that there is a need to search for materials that are resistant to more than one disease.

**Figure 11:**
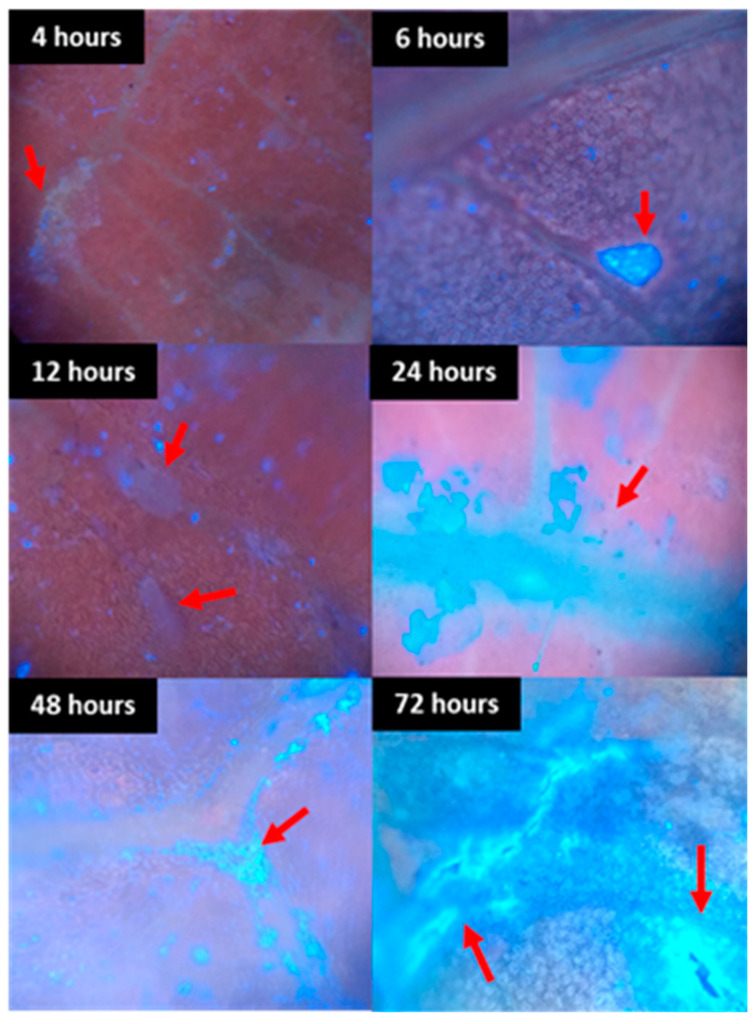
Development of *Cladosporium* spores on the surface of rubber tree leaves in UV at 25 °C subjected to different periods of wetting (4, 6, 12, 24, 48 and 72 h).

**Figure 12:**
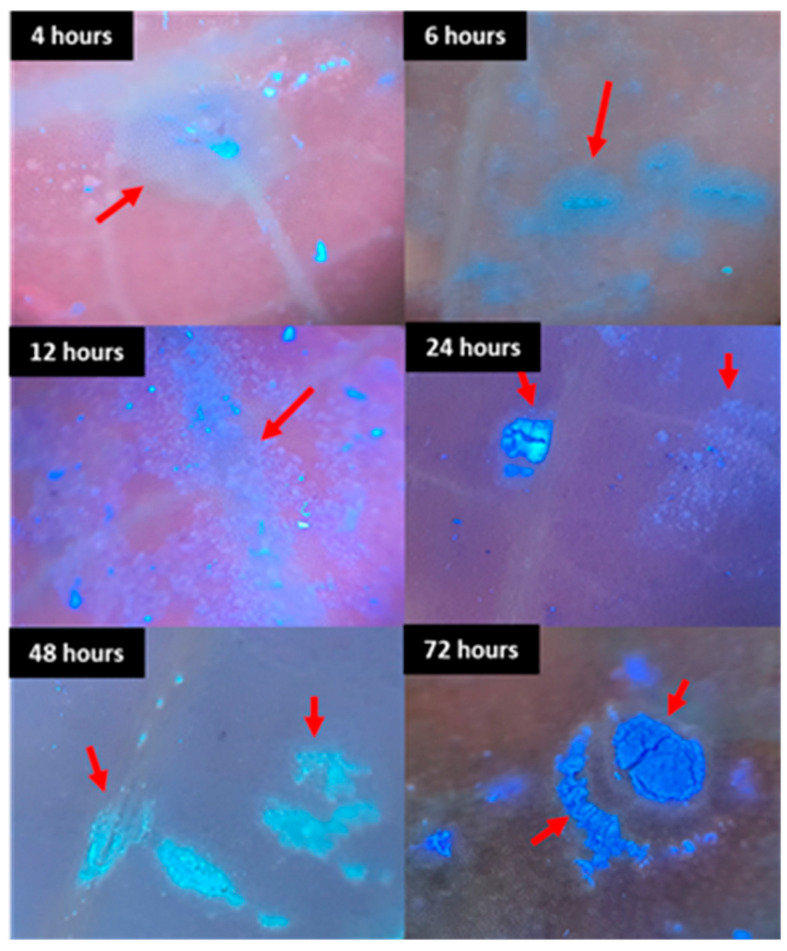
Development of sexual spores of *Davidiella* on the surface of rubber tree leaves in UV at 25 °C subjected to different periods of wetting.

As in the SEM analyses, in the UV images, it was verified that the fungus tended to develop close to leaf veins (Figure 11 and Figure 12). This is also commonly seen on the leaves of rubber trees in the field. Knowing that leaf veins are essential constituents of leaves, which play an important role in transporting plant nutrients, water, inorganic salts and trace elements [27], it is possible that the fungus has this behavior to facilitate its nutrition and survival when the plant enters the senescence phase.

## 4. Conclusions

Using microscopy techniques, it was possible to verify that the development of the fungus, both in the sexual phase, *Davidiella* sp., and in its asexual phase, *Cladosporium*, is affected by temperature, since its development is greater in temperature ranges between 20 and 25 °C. In the case of the wet period, it can also contribute to the evolution of the fungus in the plant. Thus, these initial observations improve the understanding of the fungus cycle, as there is little information about this disease in rubber trees.

## Figures and Tables

**Table 1:** Average percentage of leaf area infected by *Cladosporium* of samples under different environmental conditions.

Wetting Time (h)	Temperature (°C)						
	10	15	20	25	30	35	40
4	12. 1 Da	10.53 Ca	12.8 Da	16.19 Ea	10.31 Ca	4.0 A a	3.0 Aa
6	14.34 Cab	12.34 BCa	21.11 Db	24.81 Dbc	13.7 C a	9.0 B ab	6.64 Aa
12	16.67 Aab	22.03 CDb	23.81 Db	33.62 Ec	17.98 Bbc	18.25 Bb	16.2 A b
24	18.1 Ab	29.07 Bcd	29.31 Bc	41.27 Cd	20.5 ABc	18.77 Ab	19.55 Abc
48	30.12 Bc	39.45 Dd	38.68 De	56.24 Ede	35.34 Cd	34.08 Cc	23.0 Ac
72	45.00 Bd	55.67 De	63.24 Ef	64.3 Ee	68.0 Ee	51.87 CD d	33.54 A d
CV	32.4 *						

Average values followed by the same capital letter do not differ in the row and by the same lowercase letter in the column. * CV: coefficient of variation. Tukey test, at 5% probability, *p* > 0.05.

**Table 2:** Average percentage of leaf area infected by *Davidiella* of samples under different environmental conditions.

Wetting Time (h)	Temperature (°C)						
	10	15	20	25	30	35	40
4	4.67 BCa	4.37 Ba	6.3 Da	6.19 Da	6.64 Da	5.24 Ca	1.56 Aa
6	6.67 Ba	8.15 Cb	10.2 Db	12.1 Eb	11.8 DEbc	7.54 BCab	3.2 Aa
12	8.46 ABa	9.38 Bb	15.6 Cb	15.4 Cb	14.62 Cc	14.8 Cb	6.3 Abc
24	19.76 Bb	18.39 Bc	26.0 EDc	34.03 Ecd	28.9 Dd	18.7 Bbc	12.7 Ac
48	20.63 Bb	24.66 Cd	41.21 DEd	43.62 Ed	42.5 Ee	22.5 BCc	16.12 Acd
72	33.11 Bc	38.77 Be	44.23 Ce	49.7 Dd	50.84 Df	30.4 BCd	22.0 Ad
CV	28.7 *						

Average values followed by the same capital letter do not differ in the row and by the same lowercase letter in the column. * CV: coefficient of variation. Tukey test, at 5% probability, *p* > 0.05.

## Data Availability

The data presented in this study are available on request from the corresponding author. The data are not publicly available due to being part of a bigger project.
